# Adverse in-hospital outcomes after major cancer surgery in paraplegic patients

**DOI:** 10.1038/s41393-026-01175-4

**Published:** 2026-02-16

**Authors:** Andrea Marmiroli, Natali Rodriguez Peñaranda, Mattia Longoni, Quynh Chi Le, Fabian Falkenbach, Michele Nicolazzini, Calogero Catanzaro, Jordan A. Goyal, Stefano Luzzago, Francesco Alessandro Mistretta, Mattia Piccinelli, Fred Saad, Shahrokh F. Shariat, Alberto Briganti, Felix K. H. Chun, Salvatore Micali, Markus Graefen, Carlotta Palumbo, Riccardo Schiavina, Gennaro Musi, Pierre I. Karakiewicz

**Affiliations:** 1 Cancer Prognostics and Health Outcomes Unit, Division of Urology, University of Montréal Health Center, Montréal, QC Canada; 2 Department of Urology, IEO European Institute of Oncology, IRCCS, Via Ripamonti, 435 Milan, Italy; 3 Università degli Studi di Milano, Milan, Italy; 4 Department of Urology, Ospedale Policlinico e Nuovo Ospedale Civile S. Agostino Estense Modena, University of Modena and Reggio Emilia, Modena, Italy; 5 Vita-Salute San Raffaele University, Milan, Italy; 6 Division of Experimental Oncology/Unit of Urology, URI, Urological Research Institute, IRCCS San Raffaele Scientific Institute, Milan, Italy; 7 Goethe University Frankfurt, University Hospital, Department of Urology, Frankfurt am Main, Germany; 8 Martini-Klinik Prostate Cancer Center, University Hospital Hamburg-Eppendorf, Hamburg, Germany; 9 Piedmont, Maggiore della Carità Hospital, Novara, Italy; 10 Division of Urology, IRCCS Azienda Ospedaliero-universitaria di Bologna, Bologna, Italy; 11 Department of Oncology and Haemato-Oncology, Università degli Studi di Milano, 20122 Milan, Italy; 12 Department of Urology, Comprehensive Cancer Center, Medical University of Vienna, Vienna, Austria; 13 Department of Urology, Weill Cornell Medical College, New York, NY USA; 14 Department of Urology, University of Texas Southwestern Medical Center, Dallas, Texas USA; 15 Hourani Center for Applied Scientific Research, Al-Ahliyya Amman University, Amman, Jordan

**Keywords:** Cancer, Quality of life, Prognosis

## Abstract

**Study design:**

Observational cohort study.

**Objective:**

To test for the association between paraplegia and adverse in-hospital outcomes after major cancer surgery.

**Methods:**

Within National Inpatient Sample (2000–2019), we identified adult patients who underwent colectomy, radical hysterectomy, lung resection, gastrectomy and pancreatectomy for a primary cancer diagnosis. Descriptive analyses, propensity score matching (PSM, ratio 1:10) and multivariable logistic regression models (LRMs) were fitted.

**Setting:**

US.

**Results:**

We identified 957 (0.3%) paraplegics at colectomy, 250 (0.2%) at radical hysterectomy, 138 (0.3%) at lung resection, 94 (0.3%) at gastrectomy and 75 (0.2%) at pancreatectomy. After PSM and additional multivariable adjustment for patients, surgical and hospital characteristics, paraplegia independently predicted 12 of 12 examined endpoints after colectomy and radical hysterectomy, 11 of 12 after lung resection, 9 of 12 after pancreatectomy and 4 of 12 after gastrectomy. Across the examined surgeries, the magnitude of the increase in adverse in-hospital outcomes ranged from 2.4–3.7-fold for overall complications, 2.4–2.9-fold for intraoperative complications, 2.7–4.8-fold for vascular complications and 1.5–2.3-fold for length of stay ≥75^th^-percentile. Paraplegics also exhibited a 3.8–6.3-fold higher rate of in-hospital mortality after colectomy, lung resection and pancreatectomy, but not after gastrectomy and radical hysterectomy.

**Conclusion:**

Across the five major oncologic procedures, paraplegic patients consistently exhibited higher rates of adverse in-hospital outcomes. The excess risk was most pronounced after colectomy, radical hysterectomy, and lung resection, moderate after pancreatectomy, and least evident after gastrectomy. Similarly, the magnitude of the disadvantage also varied depending on the definition of adverse in-hospital outcome and major cancer surgery type.

## Introduction

Paraplegia may predispose to higher rates of complications and in-hospital mortality after major cancer surgery. For example, paraplegia does predispose to higher rates of adverse in-hospital outcomes after radical cystectomy and radical prostatectomy [1, 2]. However, data examining the effect of paraplegia on other major cancer surgeries are virtually nonexistent. To the best of our knowledge, individuals with paraplegia experience long-term alterations in cardiovascular, respiratory, and immune regulation, as well as higher susceptibility to infection, thromboembolic events, and impaired wound healing [3–6]. These physiological changes may place paraplegic patients at increased perioperative risk, particularly during complex oncologic procedures that require prolonged anesthesia, extensive dissection, or high blood loss. Despite these concerns, the perioperative outcomes of paraplegic patients undergoing cancer surgery have received little systematic investigation. Most prior evidence is limited to small institutional case series or non-oncologic procedures, leaving a major knowledge gap in the context of large-scale, population-based evaluation. Furthermore, recent decades have witnessed significant improvements in perioperative optimization, infection control, anesthesia safety, and postoperative rehabilitation. These advancements could potentially mitigate the differences in surgical outcomes between paraplegic and non-paraplegic individuals [7–10]. Based on this rationale, we hypothesized that adverse in-hospital outcomes would not significantly differ between paraplegic and non-paraplegic patients undergoing major cancer surgeries. To test this hypothesis, we relied on the National Inpatient Sample (NIS) database (2000–2019). We focused on five common and clinically relevant oncologic procedures—colectomy, radical hysterectomy, lung resection, gastrectomy, and pancreatectomy — chosen for their high incidence, procedural diversity, and established definitions within national surgical outcomes research [11–13].

## Methods

### Data source

To test for adverse in-hospital outcomes after major cancer surgery, we relied on discharge data from the NIS (2000–2019). The NIS is a retrospective, nationwide hospital-based administrative dataset developed for health policy and outcomes research within the Healthcare Cost and Utilization Project (HCUP) by the Agency for Healthcare Research and Quality (AHRQ) [14]. All diagnoses and procedures were coded using the International Classification of Disease (ICD) 9^th^ revision Clinical Modification (ICD-9-CM), ICD 10^th^ revision Clinical Modification (ICD-10-CM), as well as ICD 10^th^ revision Procedure Coding System (ICD-10-PCS). Ethical approval was not required for this study given the retrospective nature of the National Inpatient Sample (NIS) database and the use of publicly available, de-identified data.

### Study population

Five major cancer surgeries were selected, namely colectomy, hysterectomy, lung resection, gastrectomy and pancreatectomy, as these oncologic procedures correspond to some of the most common cancers in the general population and have not yet been comprehensively described in the existing literature [1, 2, 11–13, 15]. We focused on patients aged ≥18 years with a primary diagnosis of cancer. Specifically, we included hospitalizations of patients with colorectal (ICD-9-CM codes 153, 154 and ICD-10-CM codes C18, C19, C20), uterine (ICD-9-CM codes 178, 180, 182, 183 and ICD-10-CM codes C53, C54, C55, C56), lung and bronchus (ICD-9-CM code 162 and ICD-10-CM code C34), gastric (ICD-9-CM code 151 and ICD-10-CM code C16) or pancreatic cancer (ICD-9-CM code 157 and ICD-10-CM code C25). Video-assisted thoracoscopies were not included [16, 17].

### Definition of variables for analyses

Study endpoints included adverse in-hospital outcomes. These consisted of infectious, pulmonary, genitourinary, wound, cardiac, vascular, gastrointestinal complications and blood transfusions, length of stay ≥75^th^-percentile and of in-hospital mortality. All characteristics were identified using ICD-9 and ICD-10 codes according to previously established methodology [18, 19]. Comorbidities were defined according to Deyo modification of the Charlson Comorbidity Index (CCI) [20]. Covariates consisted of patient characteristics including age at admission (years, continuously coded), sex (female vs. male), CCI (0–1 vs. 2 vs. ≥3), as well as surgical approach (open vs. robotic) and hospital characteristics including teaching status (teaching vs. non-teaching) and size (large [≥400 beds] vs. medium [200–399 beds] vs. small [<200 beds]). Since the group of interest within the current study exclusively consisted of paraplegic patients, we tested the effect of paraplegia on adverse in-hospital outcomes without including this characteristic within the CCI calculation, according to previously reported methodology [21].

### Statistical analyses

First, propensity score matching (PSM, ratio 1:10) according to the nearest neighbor was applied to age at admission, sex, CCI, surgical approach (open vs. robotic), hospital teaching status and hospital size between paraplegic and non-paraplegic patients, to maximally reduce the effect of bias and confounders. Potential confounders were identified a priori based on clinical relevance and previous literature on perioperative outcomes in oncologic surgery. Then, multivariable logistic regression models (LRM) predicting perioperative complications and in-hospital mortality were fitted after adjustment for clustering at the hospital level using generalized estimation equation methodology [1, 2, 21–26]. All the analytical steps were first applied to patients who underwent colectomy, then applied to patients who underwent radical hysterectomy, lung resection, gastrectomy, as well as pancreatectomy. All analyses and their reporting followed the NIS reporting guidelines. Specifically, exact counts and associated proportions were not reported for samples with membership <11 [14]. All tests were two sided, with a significance level set at p < 0.05. R software environment was used for statistical computing and graphics (R version 4.2.2; R Foundation for Statistical Computing, Vienna, Austria).

## Results

### Descriptive characteristics of the study population

**Fig. 1:**
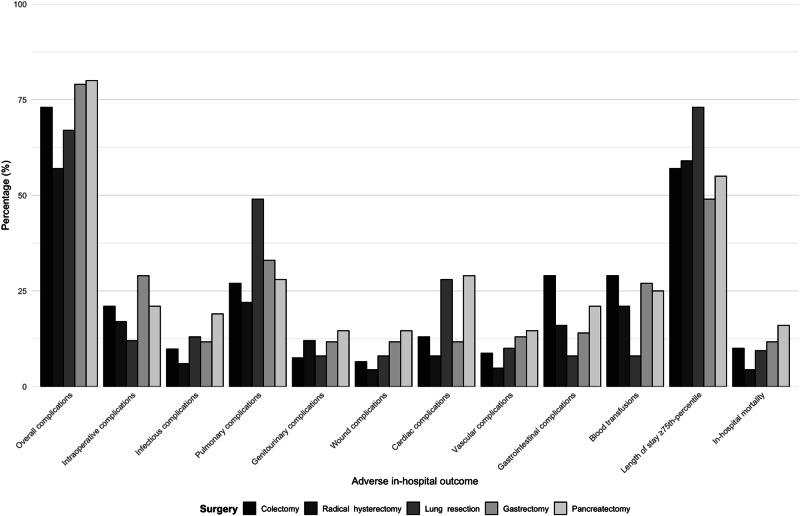
Adverse in-hospital outcomes among paraplegic patients undergoing major cancer surgeries.

Of 278,426 colorectal cancer patients treated with colectomy, 957 (0.3%) were paraplegic. Paraplegic colectomy patients were more frequently male (52 vs. 49%; p = 0.01) and more frequently harbored CCI ≥ 3 (52 vs. 43%, p < 0.001) (Table 1). After PSM, 957 of 957 (100%) paraplegic colectomy patients and 9570 of 277,469 (3.4%) non-paraplegic colectomy patients were included in further analyses. No statistically significant residual differences remained after PSM (all p-values ≥ 0.2) (Table 2). The proportions of paraplegics were 250 (0.2%) for radical hysterectomy, 138 (0.3%) for lung resection, 94 (0.3%) for gastrectomy and 75 (0.2%) for pancreatectomy (Table 1). As for colectomy, the same PSM methodology was applied in analyses examining the effect of paraplegia within the four remaining major cancer surgery types (Table 2, Fig. 1).

**Table 1:** Patients, surgery and hospital characteristics of patient undergoing major cancer surgery, namely colectomy or radical hysterectomy or lung resection or gastrectomy or pancreatectomy according to presence or absence of paraplegia, within National Inpatient Sample, between 2000 and 2019.

		Colectomy N= 278,426			Radical hysterectomy N= 109,713			Lung resection N= 53,889			Gastrectomy N= 31,635			Pancreatectomy N= 32,153		
		Paraplegic, n = 957 (0.3%)^1^	Non-paraplegic, n = 277,469(99.7%)^1^	p-value^1^	Paraplegic, n = 250 (0.2%)^1^	Non-paraplegic, n = 109,463 (99.8%)^1^	p-value^1^	Paraplegic, n = 138 (0.3%)^1^	Non-paraplegic, n = 53,751 (99.7%)^1^	p-value^1^	Paraplegic, n = 94 (0.3%)^1^	Non-paraplegic, n = 31,541 (99.7%)^1^	p-value^1^	Paraplegic, n = 75 (0.2%)^1^	Non-paraplegic, n = 32,078 (99.8%)^1^	p-value^1^
**Age at admission (Median, IQR)**		71 (61, 80)	71 (60, 79)	0.5	62 (54, 69)	61 (52, 69)	0.8	70 (64, 75)	69 (62, 75)	0.1	73 (64, 80)	68 (58, 77)	<**0.001**	69 (61, 77)	67 (59, 74)	0.2
**Sex, n** (**%)**	**Female**	457 (48.0%)	143,826(51.0%)	**0.01**	250(100%)	109,463 (100%)	-	68(49.0%)	29,516 (55.0%)	0.2	41 (44.0%)	11,964 (38.0%)	0.3	29 (39.0%)	15,997(50.0%)	0.053
	**Male**	500 (52.0%)	133,826(49.0%)		-	-		70 (51.0%)	24,235(45.0%)		53 (56.0%)	19,577 (62.0%)		46(61.0%)	16,081 (50.0%)	
**CCI,n (%)**	**0–1**	344 (36.0%)	132,238 (48.0%)		137 (55.0%)	70,280 (64.0%)		46 (33.0%)	30,027(56.0%)		29 (31.0%)	13,696(43.0%)		30 (40.0%)	14,001 (44.0%)	
	**2**	113 (12.0%)	24,965 (9.0%)	**<0.001**	24 (9.6%)	8,105 (7.4%)	**0.008**	34 (25.0%)	8,388 (16.0%)	**<0.001**	12 (13.0%)	2,757 (8.7%)	**0.03**	<11 (<14.6%)	2,977 (9.3%)	0.7
	**≥3**	500 (52.0%)	120,266 (43.0%)		89 (36.0%)	31,078 (28.0%)		58 (42.0%)	15,336 (29.0%)		53(56.0%)	15,088 (48.0%)		39 (52.0%)	15,100 (47.0%)	
**Surgical approach,n (%)**	**Open**	939 (98.0%)	271,277 (98.0%)	0.5	214(86.0%)	89,809 (82.0%)	0.1	121 (88.0%)	44,894 (84%)	0.2	91 (97.0%)	30,631 (97.0%)	0.8	72 (96.0%)	31,362 (98.0%)	0.2
	**Robotic**	18 (1.9%)	6,192 (2.0%)		36(14.0%)	19,654 (18.0%)		17 (12.0%)	8,857 (16%)		<11 (<11.7%)	910(2.9%)		<11 (<14.6%)	716 (2.2%)	
**Teaching hospital, n (%)**		477 (50.0%)	131,869 (48.0%)	0.2	199 (80.0%)	85,155 (78.0%)	0.5	109 (79.0%)	43,001 (80.0%)	0.8	54 (57.0%)	21,141(67.0%)	**0.04**	61 (81.0%)	27,406 (85.0%)	0.3
**Hospital bedsize, n (%)**	**Small**	147 (15.0%)	166,735 (60.0%)		25 (10.0%)	9,377 (8.6%)		15 (11.0%)	5,370 (10.0%)		<11 (<11.7%)	3,015 (9.6%)		<11 (<14.6%)	2,204 (6.9%)	
	**Medium**	252 (26.0%)	72,710 (26.0%)	0.3	54 (22.0%)	23,252 (21.0%)	0.7	32 (23.0%)	11,929 (22.0%)	0.9	24 (26.0%)	6,801(22.0%)	0.4	13 (17.0%)	5,512 (17.0%)	>0.9
	**Large**	558 (58.0%)	38,197 (14.0%)		171 (68.0%)	76,834 (70.0%)		91 (66.0%)	36,452 (68.0%)		64 (68.0%)	21,725 (69%)		56(75.0%)	24,362 (76.0%)	

^1^Wilcoxon rank sum test; Pearson's Chi-square test; Fisher’s exact test.

*The median age for colectomy patients: 68 years, for esophagectomy: 66 years; for hysterectomy: 62 years, for lung resection: 69 years, for pancreatectomy: 68 years.

*CCI* Charleson Comorbidity Index, *IQR* interquartile range, *n* number.

**Table 2:** Patients, surgery and hospital characteristics of patient undergoing major cancer surgery, namely colectomy or radical hysterectomy or lung resection or gastrectomy or pancreatectomy according to presence or absence of paraplegia, within National Inpatient Sample, between 2000 and 2019, after 1:10 propensity score matching.

		Colectomy N = 10,527			Radical hysterectomy N = 2,750			Lung resection N = 1,518			Gastrectomy N = 1,034			Pancreatectomy N = 825		
		Paraplegic, n = 957 (100%)^1^	Non-paraplegic, n = 9570 (3.4%)^1^	p-value^1^	Paraplegic, n = 250 (100%)^1^	Non-paraplegic, n = 2500 (2.3%)^1^	p-value^1^	Paraplegic, n = 138 (100%)^1^	Non-paraplegic, n = 1,380 (2.6%)^1^	p-value^1^	Paraplegic, n = 94 (0.3%)^1^	Non-paraplegic, n = 940 (2.9%)^1^	p-value^1^	Paraplegic, n = 75 (100%)^1^	Non-paraplegic, n = 750 (2.3%)^1^	p-value^1^
**Age at admission (Median, IQR)**		71 (61, 80)	71 (61, 80)	0.9	62(54, 69)	61 (54, 69)	>0.9	70 (64, 75)	70(64, 75)	0.8	73 (64, 80)	73 (66, 80)	0.8	69 (61, 77)	68 (60, 77)	0.7
**Sex, n (%)**	**Female**	457 (48.0%)	4,569 (48.0%)	>0.9	250 (100%)	2500 (100%)	-	68 (49.0%)	679 (49.0%)	0.8	41 (44.0%)	394 (42.0%)	0.7	29 (39.0%)	273 (36.0%)	0.7
	**Male**	500 (52.0%)	5,001 (52.0%)		-	-		70 (51.0%)	701 (51.0%)		53 (56.0%)	546 (58.0%)		46 (61.0%)	477 (64.0%)	
**CCI, n (%)**	**0–1**	344 (36.0%)	3,450 (36.0%)		137 (55.0%)	1,370 (55.0%)		46 (33.0%)	450 (33.0%)		29 (31.0%)	280 (30.0%)		30 (40.0%)	277 (37.0%)	
	**2**	113 (12.0%)	1,117 (12.0%)	>0.9	24 (9.6%)	239 (9.6%)	>0.9	34 (25.0%)	331 (24.0%)	>0.9	12 (13.0%)	114 (12.0%)	>0.9	<11 (<14.6%)	63 (8.4%)	0.9
	**≥ 3**	500 (52.0%)	5,003 (52.0%)		89 (36.0%)	891 (36.0%)		58 (42.0%)	599 (43.0%)		53 (56.0%)	546 (58.0%)		39 (52.0%)	410 (55.0%)	
**Surgical approach, n (%)**	**Open**	939 (98.0%)	9,439 (99.0%)	0.2	214 (86.0%)	2,148 (86.0%)	0.9	121 (88.0%)	1,206 (87.0%)	>0.9	91 (97.0%)	929 (99.0%)	0.1	72 (96.0%)	728 (97.0%)	0.5
	**Robotic**	18 (1.9%)	131 (1.0%)		36 (14.0%)	352 (14.0%)		17 (12.0%)	174 (13.0%)		<11 (<11.7%)	11 (1.2%)		<11 (<14.6%)	22 (2.9%)	
**Teaching hospital, n (%)**		477 (50.0%)	4,746 (50.0%)	>0.9	199 (80.0%)	1,983 (79.0%)	>0.9	109 (79.0%)	1,076 (78.0%)	0.8	54 (57.0%)	539 (57%)	>0.9	61 (81.0%)	613 (82.0%)	>0.9
**Hospital bedsize, n (%)**	**Small**	147 (15.0%)	1,456 (15.0%)		25 (10.0%)	242 (9.7%)		15 (11.0%)	128 (9.3%)		<11 (<11.7%)	59 (6.3%)		<11 (<14.6%)	59 (7.9%)	
	**Medium**	252 (26.0%)	2,497 (26.0%)	>0.9	54 (22.0%)	548 (22.0%)	>0.9	32 (23.0%)	327 (24.0%)	0.8	24 (26.0%)	229 (24.0%)	>0.9	13 (17.0%)	127 (17.0%)	>0.9
	**Large**	558 (58.0%)	5,608 (58.0%)		171 (68.0%)	1,710 (68.0%)		91 (66.0%)	925 (66.0%)		64 (68.0%)	652 (69.0%)		56 (75.0%)	564 (75.0%)	

^1^Wilcoxon rank sum test; Pearson’s Chi-square test; Fisher’s exact test; PSM for age at admission, CCI, surgical approach, teaching hospital status and hospital bedsize.

*The median age for colectomy patients: 68 years, for esophagectomy: 66 years; for hysterectomy: 62 years, for lung resection: 69 years, for pancreatectomy: 68 years.

*CCI* Charleson comorbidity index, *IQR* interquartile range; n, number.

#### Adverse in-hospital outcomes after colectomy

Adverse in-hospital outcomes after colectomy were invariably more frequent in paraplegic patients. For example, the largest absolute difference was recorded for overall complications (73 vs. 52%; Δ=21%; p < 0.001), followed by pulmonary complications (27 vs 14%; Δ=13%; p < 0.001), length of stay ≥ 75^th^-percentile (≥ 11 days) (57 vs 29%; Δ=28%; p < 0.001) and intraoperative complications (21 vs 9.7%; Δ=11.3%; p < 0.001) (Table 3). The magnitude of these differences ranged from 21 to 2.2%. In 12 out of 12 examined adverse in-hospital outcomes categories, paraplegia independently predicted 1.3-4.1-fold higher rate of adverse in-hospital outcomes (all p-values ≤ 0.002), including 1.9-fold higher rate of length of stay ≥ 75^th^-percentile (p < 0.001) and 3.8-fold higher rate of in-hospital mortality (p < 0.001) (Table 4).

**Table 3:** Adverse in-hospital outcomes of patients undergoing major cancer surgeries (colectomy, hysterectomy, lung resection, gastrectomy and pancreatectomy) according to paraplegic status within the Nationwide Inpatient Sample from 2016 to 2019 after 1:10 propensity score matching.

	Colectomy N = 10,527			Radical hysterectomy N = 2,750			Lung resection N = 1,518			Gastrectomy N = 1,034			Pancreatectomy N = 825		
Outcomes of interest	Paraplegic, n = 957 (100%)^1^	Non-paraplegic, n = 9570 (3.4%)^1^	p-value^1^ Δ	Paraplegic, n = 250 (100%)^1^	Non-paraplegic, n = 2500 (2.3%)^1^	p-value^1^ Δ	Paraplegic, n = 138 (100%)^1^	Non-paraplegic, n = 1,380 (2.6%)^1^	p-value^1^ Δ	Paraplegic, n = 94 (0.3%)^1^	Non-paraplegic, n = 940 (2.9%)^1^	p-value^1^ Δ	Paraplegic, n = 75 (100%)^1^	Non-paraplegic, n = 750 (2.3%)^1^	p-value^1^ Δ
**Overall complications**	703 (73.0%)	5,001 (52.0%)	**<0.001**Δ = 21.0	143 (57.0%)	805 (32.0%)	**<0.001** Δ = 25.0	93 (67.0%)	630 (46.0%)	**<0.001** Δ = 21.0	74 (79.0%)	559 (59.0%)	**<0.001** Δ = 20	60 (80.0%)	385 (51.0%)	**<0.001** Δ = 29.0
**Intraoperative complications**	205 (21.0%)	930 (9.7%)	**<0.001** Δ = 11.3	42 (17.0%)	195 (7.8%)	**<0.001** Δ = 9.2	17 (12.0%)	79 (5.7%)	**<0.001** Δ = 6.3	27 (29.0%)	118 (13.0%)	**<0.001** Δ = 16.0	16 (21.0%)	72 (9.6%)	**0.002** Δ = 11.4
**Postoperative complications**															
Infectious complications	94 (9.8%)	351 (3.7%)	**<0.001** Δ = 6.1	15 (6.0%)	33 (1.3%)	**<0.001** Δ = 4.7	18 (13.0%)	28 (2.0%)	**<0.001** Δ = 11.0	<11 <11.7%)	60 (6.4%)	0.7 Δ <5.3	14 (19.0%)	43 (5.7%)	**<0.001** Δ = 13.3
Pulmonary complications	259 (27.0%)	1,303 (14.0%)	**<0.001** Δ = 13	56 (22.0%)	176 (7.0%)	**<0.001** Δ = 15.0	67 (49.0%)	406 (29.0%)	**<0.001** Δ = 20.0	31 (33.0%)	251 (27.0%)	0.2 Δ = 6.0	21 (28.0%)	108 (14.0%)	**<0.001** Δ = 14.0
Genitourinary complications	72 (7.5%)	339 (3.5%)	**<0.001** Δ = 4	30 (12.0%)	124 (5.0%)	**<0.001** Δ = 7.0	11 (8.0%)	31 (2.2%)	**<0.001** Δ = 5.8	<11 <11.7%)	19 (2.0%)	>0.9 Δ <9.7	<11 (<14.6%)	19 (2.5%)	**0.02** Δ < 12.1
Wound complications	62 (6.5%)	413 (4.3%)	**0.002** Δ = 2.2	<11 (<4.4%)	41 (1.6%)	**0.04** Δ <2.8	<11 (<8.0%)	<11 (<0.8%)	**0.04** Δ < 7.2	<11 <11.7%)	56 (6.0%)	0.8 Δ <5.7	<11 (<14.6%)	48 (6.4%)	0.8 Δ < 8.2
Cardiac complications	125 (13.0%)	724 (7.6%)	**<0.001** Δ = 5.4	20 (8.0%)	89 (3.6%)	**<0.001** Δ = 4.4	39 (28.0%)	281 (20.0%)	**0.03** Δ = 8.0	<11 <11.7%)	74 (7.9%)	0.8 Δ <3.8	22 (29.0%)	81 (11.0%)	**<0.001** Δ = 18.0
Vascular complications	83 (8.7%)	221 (2.3%)	**<0.001** Δ = 6.4	12 (4.8%)	46 (1.8%)	**0.002** Δ = 3.0	14 (10.0%)	38 (2.8%)	**<0.001** Δ = 7.2	12 (13.0%)	28 (3.0%)	**<0.001** Δ = 10.0	<11 (<14.6%)	33 (4.4%)	**0.003** Δ <10.2
Gastrointestinal complications	280 (29.0%)	2,353 (25.0%)	**0.001** Δ = 4.0	41 (16.0%)	258 (10.0%)	**0.003** Δ = 6.0	<11 (<8.0%)	23 (1.7%)	0.2 Δ < 6.3	13 (14.0%)	135 (14.0%)	0.9 Δ = **0**	16 (21.0%)	108 (16.0%)	0.1 Δ = 5.0
**Blood transfusions**	281 (29.0%)	1,920 (20.0%)	**<0.001** Δ = 9.0	52 (21.0%)	322 (13.0%)	**<0.001** Δ = 8.0	11 (8.0%)	59 (4.3%)	**0.04** Δ = 3.7	25 (27.0%)	271 (29.0%)	0.6 Δ = 2.0	19 (25.0%)	145 (19.0%)	0.2 Δ = 6.0
**Length of stay ≥ 75**^**th**^-percentile*	546 (57.0%)	2,782 (29.0%)	**<0.001** Δ = 28.0	148 (59.0%)	813 (33.0%)	**<0.001** Δ = 26.0	101 (73.0%)	428 (31.0%)	**<0.001** Δ = 42.0	46 (49.0%)	304 (32.0%)	**0.001** Δ = 17.0	41 (55.0%)	196 (26.0%)	**0.002**Δ = 29.0
**In-hospital mortality**	99 (10.0%)	290 (3.0%)	**<0.001** Δ = 7.0	<11 (<4.4%)	<11 (<0.4%)	**<0.001** Δ <4.0	13 (9.4%)	25 (1.8%)	**<0.001** Δ = 7.6	<11 <11.7%)	70 (7.4%)	0.3 Δ <4.3	12 (16.0%)	25 (3.3%)	**<0.001** Δ = 12.7

^1^Wilcoxon rank sum test; Pearson’s Chi-square test; Fisher’s exact test; Δ quantify the absolute difference between rates in non paraplegic vs. paraplegic patients; PSM for age at admission, sex, CCI, surgical approach, teaching hospital status and hospital bedsize.

*Length of stay ≥ 75th-percentile: for colectomy: 11 days; for hysterectomy: 5 days; for lung resection: 7 days, for gastrectomy: 15 days, for pancreatectomy: 14 days.

*CCI* Charleson comorbidity index, *IQR* interquartile range, *n* number.

**Table 4:** Multivariable logistic regression models (LRM) predicting the effect of paraplegia status on 12 adverse in-hospital outcomes categories after major cancer surgery, namely colectomy, radical hysterectomy, lung resection, gastrectomy and pancreatectomy, after adjustment for clustering at the hospital level using generalized estimation equation (GEE) methodology and after 1:10 propensity score matching.

		Colectomy N = 10,527						Radical hysterectomy N = 2750					Lung resection N = 1518						Gastrectomy N = 1034						Pancreatectomy N = 825					
Outcomes of interest		OR	(95% CI)			p-value^1^		OR	(95% CI)			p-value^1^	OR		(95% CI)		p-value^1^		OR		(95% CI)		p-value^1^		OR		(95% CI)		p-value^1^	
**Overall complications**	2.5			(2.1, 2.9)	**<0.001**		3.0			(2.3, 4.0)	**<0.001**			2.4		(1.6, 3.4)		**<0.001**		2.5		(1.5, 4.1)		**<0.001**		3.7		(1.9, 6.8)		**<0.001**
**Intraoperative complications**	2.6			(2.1, 3.0)	**<0.001**		2.4			(1.7, 3.4)	**<0.001**			2.4		(1.3, 4.2)		**0.003**		2.9		(1.8, 4.7)		**<0.001**		2.6		(1.5, 4.8)		**0.002**
**Postoperative complications**																														
Infectious complications	2.9			(2.3, 3.7)	**<0.001**		5.0			(2.6, 9.4)	**<0.001**			7.4		(3.9, 13.7)		**<0.001**		0.8		(0.3, 2.1)		0.7		3.9		(2.0, 7.8)		**<0.001**
Pulmonary complications	2.4			(2.1, 2.8)	**<0.001**		4.1			(2.9, 5.7)	**<0.001**			2.1		(1.5, 3.1)		**<0.001**		1.4		(0.9, 2.2)		0.1		2.4		(1.4, 4.2)		**0.002**
Genitourinary complications	2.2			(1.7, 2.9)	**<0.001**		2.7			(1.7, 4.1)	**<0.001**			3.7		(1.8, 7.5)		**<0.001**		1.0		(0.2, 4.9)		0.9		3.5		(1.2, 9.7)		**0.01**
Wound complications	1.5			(1.2, 2.0)	**0.002**		2.3			(1.1, 4.7)	**0.03**			11.5		(1.4, 91.1)		**0.02**		1.1		(0.4, 2.5)		0.9		1.2		(0.5, 2.9)		0.7
Cardiac complications	1.8			(1.5, 2.3)	**<0.001**		2.5			(1.4, 4.2)	**<0.001**			1.6		(1.1, 2.3)		**0.02**		1.1		(0.5, 2.3)		0.8		4.4		(2.4, 8.1)		**<0.001**
Vascular complications	4.1			(3.1, 5.2)	**<0.001**		2.7			(1.4, 5.2)	**0.003**			4.3		(2.3, 8.2)		**<0.001**		4.8		(2.4, 9.9)		**<0.001**		3.5		(1.6, 7.6)		**0.002**
Gastrointestinal complications	1.3			(1.1, 1.5)	**0.002**		1.7			(1.2, 2.6)	**0.006**			2.2		(0.8, 6.0)		0.1		0.9		(0.5, 1.8)		0.9		1.6		(0.9, 2.8)		0.1
**Blood transfusions**	1.7			(1.5, 2.0)	**<0.001**		1.8			(1.3, 2.5)	**<0.001**			1.9		(1.0, 3.9)		**0.04**		0.9		(0.6, 1.5)		0.8		1.4		(0.8, 2.5)		0.3
Length of stay ≥ 75th-percentile^*^	1.9			(1.9, 2.1)	**<0.001**		1.8			(1.6, 2.0)	**<0.001**			2.3		(2.1, 2.7)		**<0.001**		1.5		(1.2, 1.9)		**<0.001**		2.1		(1.6, 2.6)		**<0.001**
**In-hospital mortality**	3.8			(3.0, 4.9)	**<0.001**		NA			NA	NA			5.8		(2.9, 11.6)		**<0.001**		1.5		(0.7, 3.1)		0.2		6.3		(2.8, 13.5)		**<0.001**

^1^Wilcoxon rank sum test; Pearson’s Chi-square test; Fisher’s exact test; Δ quantify the absolute difference between rates in non paraplegic vs. paraplegic patients; PSM for age at admission, sex, CCI, surgical approach, teaching hospital status and hospital bedsize.

*Length of stay ≥ 75th-percentile: for colectomy: 11 days; for hysterectomy: 5 days; for lung resection: 7 days, for gastrectomy: 15 days, for pancreatectomy: 14 days.

*CCI* Charleson Comorbidity Index, *IQR* interquartile range, *n* number, *OR* odds ratio.

#### Adverse in-hospital outcomes after radical hysterectomy

Adverse in-hospital outcomes after radical hysterectomy followed virtually the same pattern as after colectomy. Specifically, paraplegic patients invariably exhibited higher rates of length of stay ≥ 75^th^-percentile (≥ 5 days) (59 vs 33%; Δ=26%; p < 0.001), overall complications (57 vs. 32%; Δ=25%; p < 0.001) and pulmonary complications (22 vs 7%; Δ=15%; p < 0.001) (Table 3). The magnitude of the differences ranged from 26 to <2.8%. In 12 out of 12 examined adverse in-hospital outcomes categories, paraplegia independently predicted 1.5 – 5.0-fold higher rate of adverse in-hospital outcomes (all p-values ≤ 0.03), including 1.8-fold higher rate of length of stay ≥ 75^th^-percentile (p < 0.001). Due to the low number of events, radical hysterectomy was excluded from the calculation of in-hospital mortality relative rates (Table 4).

#### Adverse in-hospital outcomes after lung resection

Adverse in-hospital outcomes after lung resection were comparable to colectomy and radical hysterectomy. However, higher rates of adverse in-hospital outcomes were recorded in 11 out of 12 examined categories. The absolute differences and relative differences were similar. Specifically, the magnitude of the absolute differences ranged from 42–3.7%. Additionally, paraplegia independently predicted 1.6-11-fold higher rate of adverse in-hospital outcomes (all p-values ≤ 0.04), including 2.3-fold higher rate of length of stay ≥ 75^th^-percentile (≥7 days) (p < 0.001) and 5.8-fold higher rate of in-hospital mortality (p < 0.001). Conversely, paraplegia was not an independent predictor of gastrointestinal complications (p = 0.1) (Table 4).

#### Adverse in-hospital outcomes after gastrectomy

Adverse in-hospital outcomes after gastrectomy were more frequent in paraplegic patients, however the absolute and relative differences applied to only four out of 12 examined categories. Specifically, paraplegic patients exhibited higher rates of overall complications (79 vs. 59%, Δ=20%; p < 0.001), length of stay ≥75^th^-percentile (≥14 days) (49 vs 32%; Δ=17%; p = 0.001), intraoperative complications (29 vs. 13%; Δ=16%; p < 0.001) and vascular complications (13 vs 3%; Δ=10%; p < 0.001) (Table 3). The magnitude of absolute differences ranged from 20 to 10%. Additionally, paraplegia independently predicted 1.5 – 4.8-fold higher rate of adverse in-hospital outcomes (all p-values < 0.001), including 1.5-fold higher rate of length of stay ≥75^th^-percentile, but not higher rate of in-hospital mortality (Table 4).

#### Adverse in-hospital outcomes after pancreatectomy

Adverse in-hospital outcomes were more frequent in paraplegic patients after pancreatectomy. However, the absolute and relative differences applied to only nine out of 12 examined categories. Specifically, paraplegic patients exhibited higher rates of overall complications (80 vs. 51%; Δ=29%; p < 0.001), length of stay ≥75^th^-percentile (≥15 days) (55 vs 26%; Δ=29%; p = 0.002) and cardiac complications (29 vs 11%; Δ=18%; p < 0.001). The absolute differences ranged from 29 to <10.2%. (Table 3). Additionally, paraplegia independently predicted 2.1 – 6.3-fold higher rate adverse in-hospital outcomes (all p-values ≤ 0.01), including 2.1-fold higher rate of length of stay ≥75^th^-percentile (p < 0.001) and 6.3-fold higher rate of in-hospital mortality (p < 0.001). (Table 4).

## Discussion

The association between paraplegia and adverse in-hospital outcomes after major cancer surgeries is virtually unknown. To address this knowledge gap, we relied on a large-scale population-based cohort within the NIS (2000–2019) testing the effect of paraplegia between paraplegic vs. non-paraplegic colectomy, radical hysterectomy, lung resection, gastrectomy and pancreatectomy patients. We made several noteworthy observations as follows.

First, the association between paraplegia and adverse in-hospital outcomes was never specifically and systematically addressed in large scale studies. In consequence, it was unknown to what extent paraplegic patients undergo major cancer surgeries. The current study demonstrated that a very small proportion of patients treated with major cancer surgeries are paraplegics. These rates ranged from 0.2 to 0.3% across the five examined major cancer surgery types. Despite the extremely large sample size available in the current database, the absolute numbers of paraplegic patients within one of the five major cancer surgery types, ranged from elevated at colectomy (n = 957) to relatively low at pancreatectomy (n = 75). These observations indicate the difficulty in systematic assessment of paraplegia effect on various endpoints in surgical patients.

Second, aside from paraplegia that distinguished the cases from non-paraplegic controls, we only identified one important difference in baseline characteristics between these two patient groups, namely CCI. Specifically, even after exclusion of paraplegia within CCI definition, these individuals harbored a higher count than their non-paraplegic counterparts. Interestingly, the magnitude of the difference in CCI was more pronounced at lung resection (42 vs. 29%, p < 0.001), followed by colectomy (52 vs. 43%, p < 0.001), radical hysterectomy (36 vs. 28%, p = 0.008) and gastrectomy (56 vs. 48%, p = 0.03). Additionally, no statistically significant difference in CCI count was identified at pancreatectomy (p = 0.7). Beside CCI, age at admission, sex and teaching hospital status exhibited statistically significant differences that may not be considered as clinically meaningful. However, their contribution requires consideration in statistical testing. In consequence, PSM was applied to maximally reduce or ideally eliminate the source of bias.

Third, after relying on PSM and multivariable adjustment within each of the five examined major cancer surgeries, paraplegia was invariably associated with higher rates of adverse in-hospital outcomes that ranged from twelve to as few as four examined categories. Similarly, the magnitude of the effect associated with paraplegia also ranged from 11-fold to only 1.3-fold increase. Most pronounced effect was recorded after colectomy and radical hysterectomy where paraplegia affected all twelve examined adverse in-hospital outcome categories. Conversely, increases in adverse in-hospital outcomes were respectively recorded in eleven, nine and four endpoints after lung resection, pancreatectomy and gastrectomy. These observations indicate that paraplegia exerts a specific effect on a distinguished number of adverse in-hospital outcomes and its magnitude is also specific for each of the examined major cancer surgery types. In consequence, major cancer surgery types-specific analyses are required. Moreover, a systematic approach such as the one used in the current study is clearly needed to address and quantify the association between paraplegia and adverse in-hospital outcomes according to specific adverse in-hospital outcomes definition.

Fourth, we also examined in-hospital mortality since it represents the most dire potential complication. Here, paraplegic patients invariably exhibited higher rates of in-hospital mortality than their non-paraplegic counterparts. Specifically, paraplegic patients exhibited higher rates of in-hospital mortality after pancreatectomy (16.0% vs. 3.3%; p < 0.001), lung resection (9.4% vs. 1.8%; p = 0.001), colectomy (10% vs. 3%; p < 0.001) and radical hysterectomy (< 4.4% vs. <0.4%; p < 0.001), but not after gastrectomy (p = 0.3). In multivariable analyses, presence of paraplegia independently predicted 3.8-, 5.8- and 6.3-fold higher rates of in-hospital mortality, for colectomy, lung resection and pancreatectomy, respectively. These observations are essential to communicate to paraplegic patients at clinical decision-making and informed consent.

Fifth, we also quantified the effect of paraplegia on length of stay ≥75^th^-percentile. Expectedly, a larger proportion of paraplegic patients required length of stay ≥75^th^-percentile. Interestingly, the magnitude of the increase was not related to the absolute length of stay ≥75^th^-percentile. After multivariable adjustment, the magnitude of the differences between length of stay ≥75^th^-percentile was greatest after lung resection (absolute: 7 days; OR 2.3), followed by pancreatectomy (absolute: 14 days; OR 2.1), colectomy (absolute: 11 days; OR 1.9), radical hysterectomy (absolute: 5 days; OR 1.8) and gastrectomy (absolute: 15 days; OR 1.5). In consequence, similarly to other examined endpoints, the effect of paraplegia differs according to major cancer surgery types when length of stay ≥75^th^-percentile represents the endpoints of interest.

Sixth, several mechanisms may explain the excess perioperative morbidity observed in paraplegic patients. Beyond the intrinsic autonomic, cardiovascular, and immune dysregulation associated with paraplegia [27–29], secondary factors such as neurogenic bladder and bowel dysfunction, reduced mobility, and impaired skin integrity contribute to urinary tract infections, ileus, and pressure ulcers—complications that may be partially preventable with specialized care [28–31]. In many general hospitals performing major cancer surgeries, expertise and resources specific to paraplegics’ management are limited. Inadequate bowel/bladder management, suboptimal positioning, and delayed mobilization may thus aggravate postoperative risk [30, 31]. Multidisciplinary co-management or early referral to specialized SCI centers—where structured nursing protocols and rehabilitation support are routine—could mitigate these risks [31, 32]. Where referral is not feasible, targeted education of surgical and nursing staff in bowel, bladder, and skin care protocols may represent a practical alternative. Future studies should examine whether such institutional factors modify perioperative outcomes in paraplegic surgical patients [33].

Taken together, the current observations indicate that paraplegic patients are invariably at higher risk of adverse in-hospital outcomes. The extent and the magnitude of the differences in adverse in-hospital outcomes, length of stay ≥75^th^-percentile and mortality rates are specific to each of the five examined major cancer surgeries.

The current study provides a first systematic assessment of the effect of paraplegia on 12 different adverse in-hospital outcomes and should ideally be used for medical decision-making at informed consent prior to one of the five examined major cancer surgeries. Despite the novelty of our observations, the present study is not devoid of limitations. First, the current results are only applicable to the five examined major cancer surgeries. They may not be extrapolated to other surgeries that may or may not be oncological. Second, selection and reporting biases may have remained due to the retrospective nature of the NIS. This limitation applies to the current study as well as to all previous analyses relying on the NIS [10–17] or other large-scale retrospective databases, such as the Surveillance Epidemiology and End Results database. Third, despite the very large size of the NIS, paraplegic patients were rare. Due to NIS reporting limitations, specific details could not be provided for some of the comparisons when patients count were less than 11. Instead, only relative metrics, such as OR, could be provided. Additionally, the amount of details included in the current analysis was also limited due to the nature of the NIS. For example, the specific etiology and clinical presentation of paraplegia and its duration, as well as the level of spinal cord injury were not available. Additionally, the NIS lacks relevant clinical data such as cancer stage, mean follow-up, neoadjuvant therapies, emergent versus elective status, and functional status. Furthermore, the small sample size for certain procedures (e.g., gastrectomy and pancreatectomy) precludes general causal conclusions. Moreover, despite the use of a standardized approach, a limited amount of details was available regarding the nature of the complications that were examined. For example, the amount of blood units used in transfusions was not known. Specifically, all of the complications examined within the current database were recorded retrospectively. Last but not least, the NIS exclusively provides in-hospital data. Therefore, it was not possible to assess further complications after the patient was discharged after major cancer surgery. Nevertheless, the present study provides the most detailed and methodologically structured analysis on the association between paraplegia and adverse in-hospital outcomes in paraplegic major cancer surgery patients.

### Conclusion

Across the five major oncologic procedures, paraplegic patients consistently exhibited higher rates of adverse in-hospital outcomes. The excess risk was most pronounced after colectomy, radical hysterectomy, and lung resection, moderate after pancreatectomy, and least evident after gastrectomy. Similarly, the magnitude of the disadvantage also varied depending on the definition of adverse in-hospital outcome and major cancer surgery type.

## Supplementary information

STROBE CHECKLIST

## Data Availability

The data that support the findings of this study are available from the Healthcare Cost and Utilization Project (HCUP) National Inpatient Sample (NIS). Restrictions apply to the availability of these data, which were used under license for the current study and are not publicly available. Data are available from HCUP upon reasonable request and completion of the required Data Use Agreement.
