# Supplementary material for: Adverse in-hospital outcomes after major cancer surgery in paraplegic patients
Source: Spinal Cord. 2026 Feb 16;64(4):362–70. doi: 10.1038/s41393-026-01175-4 (PMC13065474; doi:10.1038/s41393-026-01175-4)
Supplement: Supplementary file 1 — STROBE CHECKLIST [file 41393_2026_1175_MOESM1_ESM.docx]

# STROBE Statement—Checklist of items that should be included in reports of cohort studies

Study: Adverse In-Hospital Outcomes After Major Cancer Surgery in Paraplegic Patients

| Section | Item No | Recommendation | Included? | Comments |
| --- | --- | --- | --- | --- |
| Title and abstract | 1(a) | Indicate the study’s design with a commonly used term in the title or the abstract | Yes | The abstract specifies 'Observational cohort study'. Clear and appropriate. |
|  | 1(b) | Provide in the abstract an informative and balanced summary of what was done and what was found | Yes | Abstract includes objectives, methods (PSM, LRM), and key results. |
| Introduction | 2 | Explain the scientific background and rationale for the investigation being reported | Yes | Background and rationale clearly explained (knowledge gap on paraplegia in oncologic surgery). |
|  | 3 | State specific objectives, including any prespecified hypotheses | Yes | Objective clearly stated: to test the association between paraplegia and adverse in-hospital outcomes. |
| Methods | 4 | Present key elements of study design early in the paper | Yes | Observational cohort design explicitly stated. |
|  | 5 | Describe the setting, locations, and relevant dates, including periods of recruitment, exposure, follow-up, and data collection | Yes | NIS database, 2000–2019, described as data source. |
|  | 6(a) | Give the eligibility criteria, and the sources and methods of participant selection. Give the rationale for the choice of participants | Yes | Inclusion: adults ≥18 with primary cancer diagnosis undergoing one of five major surgeries. |
|  | 6(b) | For matched studies, give matching criteria and the number of controls per case | Yes | PSM 1:10 ratio by age, sex, comorbidity, surgical and hospital variables. |
|  | 7 | Clearly define all outcomes, exposures, predictors, potential confounders, and effect modifiers | Yes | 12 outcome categories defined; covariates described in Methods section. |
|  | 8 | For each variable of interest, give sources of data and details of methods of assessment | Yes | ICD-9/ICD-10 codes used and referenced. |
|  | 9 | Describe any efforts to address potential sources of bias | Partially | Bias minimized via PSM and multivariable regression; residual confounding acknowledged. |
|  | 10 | Explain how the study size was arrived at | Yes | All eligible NIS records were included; no sample size calculation needed. |
|  | 11 | Explain how quantitative variables were handled in the analyses | Yes | Continuous variables (age) modeled directly; CCI categorized (0–1, 2, ≥3). |
|  | 12(a) | Describe all statistical methods, including those used to control for confounding | Yes | PSM and multivariable logistic regression with hospital-level clustering. |
|  | 12(b) | Describe any methods used to examine subgroups and interactions | Partially | Analyses stratified by surgery type; no explicit interaction testing. |
|  | 12(c) | Explain how missing data were addressed | Partially | Missing data not described; likely negligible in NIS. Could be added explicitly. |
|  | 12(d) | If applicable, explain how loss to follow-up was addressed | N/A | NIS captures in-hospital events only; no follow-up. |
|  | 12(e) | Describe any sensitivity analyses | No | Not reported; could mention consistency between PSM and LRM results as robustness check. |
| Results | 13(a) | Report numbers of individuals at each stage of study | Yes | Number of eligible and matched participants for each surgery type reported. |
|  | 13(b) | Give reasons for non-participation at each stage | Partially | No exclusions mentioned; could specify if records were omitted for incomplete data. |
|  | 14(a) | Give characteristics of study participants and information on exposures and potential confounders | Yes | Baseline characteristics detailed in Tables 1–2. |
|  | 14(b) | Indicate number of participants with missing data for each variable | No | Not reported explicitly. |
|  | 15 | Report outcome data for each exposure group | Yes | Reported per surgery type (Tables 3–4). |
|  | 16(a) | Give unadjusted and adjusted estimates with precision (e.g., 95% CI) | Yes | Unadjusted and adjusted ORs reported; CIs and p-values provided. |
|  | 16(b) | Report category boundaries when continuous variables were categorized | Yes | CCI and LOS ≥75th percentile defined clearly. |
|  | 16(c) | If relevant, translate relative risk into absolute risk | Yes | Absolute and relative differences provided in Results. |
|  | 17 | Report other analyses (subgroups, interactions, sensitivity) | Partially | Subgroup by surgery type only; no sensitivity analyses reported. |
| Discussion | 18 | Summarise key results with reference to study objectives | Yes | Main findings summarized clearly in Discussion and Conclusion. |
|  | 19 | Discuss limitations of the study | Yes | Comprehensive discussion of retrospective design, missing variables, limited sample size. |
|  | 20 | Give cautious overall interpretation of results | Yes | Interpretation balanced and consistent with objectives and evidence. |
|  | 21 | Discuss generalisability of results | Yes | Results generalizable to similar major oncologic surgeries in paraplegic patients. |
| Other information | 22 | Give source of funding and role of funders | Yes | No funding; conflicts of interest declared absent. |
